# Gastric-Type Mixed Neoplastic and Non-Neoplastic Lesions in the Duodenal Bulb: A Case Supporting the Metaplasia–Neoplasia Sequence

**DOI:** 10.3390/diagnostics16071045

**Published:** 2026-03-30

**Authors:** Hidetoshi Satomi, Noriya Uedo, Shingo Ishiguro, Yoshiki Kairiku, Tomoki Michida, Ryu Ishihara, Keiichiro Honma

**Affiliations:** 1 Department of Diagnostic Pathology and Cytology, Osaka International Cancer Institute, 3-1-69 Otemae, Osaka 541-8567, Japan; keiichirou.honma@oici.jp; 2 Department of Gastrointestinal Oncology, Osaka International Cancer Institute, Osaka 540-0008, Japan; noriya.uedo@gmail.com (N.U.); earth1003@gmail.com (Y.K.); tmichida3@gmail.com (T.M.); ryu1486@gmail.com (R.I.); 3 PCL Osaka Pathology & Cytology Center, Osaka 533-0031, Japan; shingo_ishiguro@bml.co.jp

**Keywords:** pyloric gland adenoma, duodenum, gastric foveolar metaplasia, hyperplastic polyp, metaplasia–neoplasia sequence, field carcinogenesis

## Abstract

Gastric-type lesions in the duodenum, including pyloric gland adenoma and gastric foveolar metaplasia, have been increasingly recognized for their unique histogenesis and potential link through the metaplasia–neoplasia sequence. However, the coexistence of neoplastic and non-neoplastic gastric-type lesions within the same histological section has not been previously reported. Here, we present a case of a 73-year-old Japanese woman who underwent endoscopic submucosal dissection for a 34 × 20 mm elevated lesion in the duodenal bulb. Based on the preoperative biopsy results, pyloric gland adenoma was diagnosed; however, histopathological examination of the resected specimen revealed a far more complex picture. The main lesion consisted of two contiguous components: a hyperplastic polyp with gastric foveolar-type phenotype (Lesion I) and a pyloric gland adenoma mixed with gastric foveolar-type hyperplastic polyp (Lesion II). Importantly, the transitional zone between these components demonstrated histological continuity, with areas showing admixture of hyperplastic and adenomatous features within the same microscopic field. A separate hyperplastic polyp with gastric foveolar-type phenotype (Lesion III) was also identified, separated from Lesions I and II by intervening normal duodenal mucosa. All lesions shared a gastric-type mucin phenotype (MUC5AC-positive, CD10-negative), and extensive Brunner’s gland hyperplasia was observed throughout the specimen. This case provides compelling morphological evidence for a histogenetic link between non-neoplastic gastric-type hyperplasia and pyloric gland adenoma, supporting the concept of a metaplasia–neoplasia sequence in the duodenum. Furthermore, the presence of an additional separate lesion with the same phenotype suggests a field change in the development of gastric-type lesions.

## 1. Introduction

Duodenal polyps are relatively uncommon lesions; however, they are being increasingly detected with the widespread use of upper gastrointestinal endoscopy and awareness of the condition [1]. These “polyps” are a histologically diverse group of lesions, including non-neoplastic lesions such as Brunner’s gland hyperplasia, hyperplastic polyps, and inflammatory polyps, as well as neoplastic lesions such as adenomas. Among these, gastric-type lesions that are characterized by gastric foveolar or pyloric gland differentiation have attracted increasing attention due to their unique histogenesis and potential clinical significance.

Pyloric gland adenoma (PGA) is a rare but distinctive neoplasm that typically arises in the stomach but can also occur in extragastric sites, including the duodenum [2]. Despite its bland histological appearance, PGA is recognized as a true neoplasm with potential to become malignant [3]. In addition to PGA, non-neoplastic gastric-type lesions such as gastric foveolar metaplasia (GFM) and hyperplastic polyps with gastric-type phenotype have been detected in the duodenum, typically in association with mucosal injury or *Helicobacter pylori* infection [1].

The histogenesis of these gastric-type lesions in the duodenum has been linked to the “neo-G zone” hypothesis, which proposes that mucosal injury induces regenerative changes in Brunner’s glands, resulting in gastric-type epithelium [4]. Furthermore, recent molecular studies have revealed that both neoplastic and non-neoplastic gastric-type lesions in the duodenum share common genetic alterations, including *GNAS* and *KRAS* mutations, suggesting a possible link between gastric-type metaplasia and subsequent neoplastic transformation [5]. However, the coexistence of neoplastic and non-neoplastic gastric-type lesions within the same histological section has rarely been reported.

Herein, we report a rare case of PGA and hyperplastic polyp components with a gastric foveolar-type phenotype that coexisted as contiguous lesions within a single duodenal specimen. Notably, the transitional zone demonstrated histological continuity between the neoplastic and non-neoplastic components. To our knowledge, this is the first report documenting such a coexistence and histological transition between PGA and a hyperplastic polyp within the same lesion. This case provides compelling morphological evidence supporting the metaplasia–neoplasia sequence in the development of gastric-type duodenal neoplasms.

## 2. Case Presentation

A 73-year-old Japanese woman was referred to our hospital after an elevated lesion was detected in the duodenal bulb during a screening esophagogastroduodenoscopy. Her medical history was significant only for hypertension. She had no family history of gastrointestinal malignancies or polyposis syndromes and was asymptomatic at the time of presentation.

**Figure 1:**
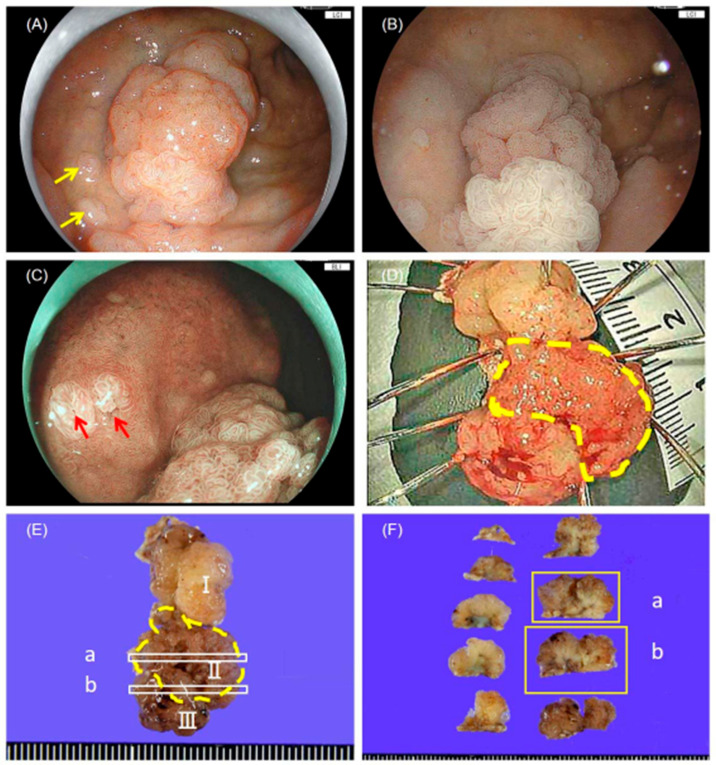
Endoscopic and gross findings. (**A**) White-light endoscopy showing a 34 × 20 mm elevated lesion with a granular surface in the duodenal bulb. The central portion appeared slightly redder than the surrounding areas. Small nodules were observed in the surrounding mucosa (yellow arrows). (**B**) Narrow-band imaging (NBI) of the main lesion demonstrated enlarged microsurface structures. (**C**) NBI revealed that the small nodules had enlarged microsurface structures with central orifices (red arrows), suggesting Brunner’s gland hyperplasia with gastric foveolar metaplasia. (**D**) The freshly resected specimen showed a lobulated, elevated appearance. The central segment (yellow dotted line) had a finely granular surface with prominent redness. (**E**) The formalin-fixed specimen showed a sessile elevated lesion with a lobulated appearance. The cutting lines for slices “a” and “b” are indicated. Lesions I, II, and III are labeled. (**F**) Cross-sections of the formalin-fixed specimen cut along the lines indicated in (**E**). Slice “a” (yellow box, upper right) contained Lesions I, II, and III, and slice “b” (yellow box, lower right) contained Lesions II and III.

Esophagogastroduodenoscopy (GIF-XZ1200 and GIF-H290T; Olympus, Tokyo, Japan) revealed a 34 × 20 mm elevated lesion in the duodenal bulb (Figure 1A–C). The lesion appeared as a single polypoid mass endoscopically (Figure 1A). Narrow-band imaging (NBI) demonstrated enlarged microsurface structures in the main lesion (Figure 1B), and the small nodules showed features suggestive of Brunner’s gland hyperplasia with GFM (Figure 1C). Endoscopic examination of the stomach indicated atrophic gastritis. A biopsy was performed prior to referral, and histological examination, including re-evaluation at our institution, yielded a diagnosis of PGA. Given the size of the lesion, the neoplastic biopsy diagnosis, and the heterogeneous endoscopic appearance, endoscopic submucosal dissection (ESD) was performed for complete removal of the lesion and definitive histological diagnosis.

The main ESD specimen measured 34 × 20 mm and demonstrated a sessile elevated lesion with a lobulated appearance (Figure 1D–F). Upon sectioning, three areas were identified and designated as Lesions I, II, and III for descriptive purposes (Figure 1E), although Lesions I and II were contiguous components of the main lesion.

**Figure 2:**
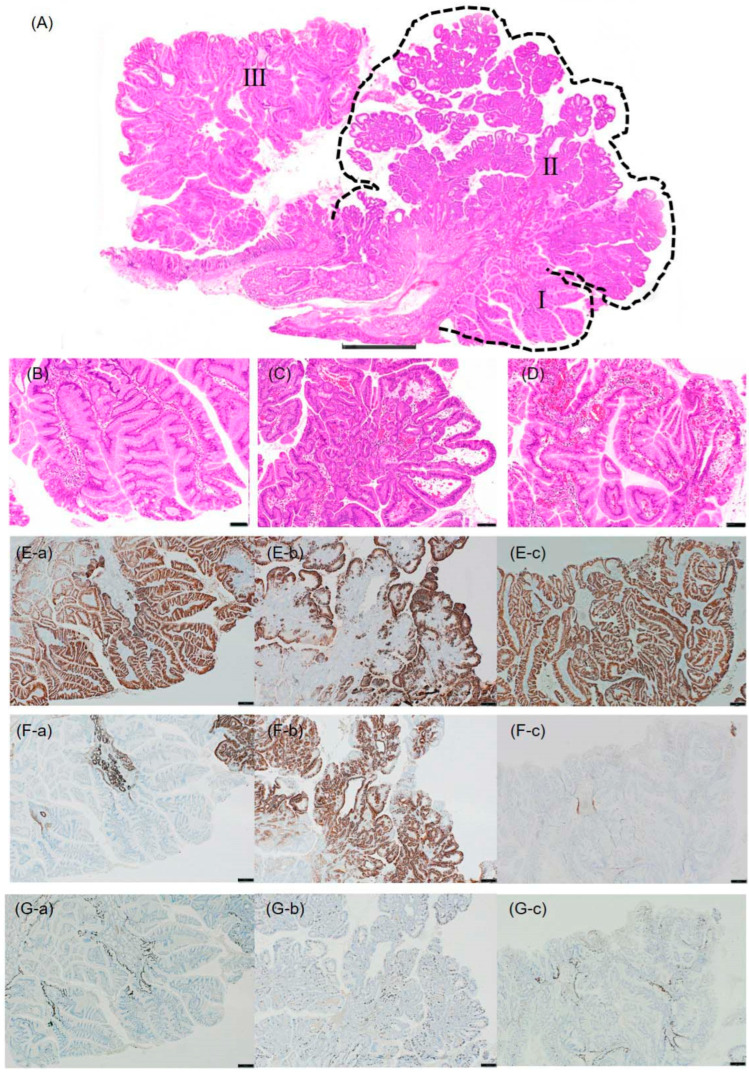
Histological and immunohistochemical findings of slice “a” containing Lesions I, II, and III. (**A**) Loupe image showing the distribution of all three lesions within the same histological section. Lesion I (lower right, dashed line), Lesion II (upper right, dashed line), and Lesion III (upper left) are indicated. Note that Lesions I and II are contiguous, whereas Lesion III is separated by intervening normal mucosa. Scale bar: 2 mm. (**B**) High magnification view of Lesion I: columnar epithelial cells with pale cytoplasm and mild nuclear atypia. Scale bar: 100 μm. (**C**) High magnification view of Lesion II: atypical cells with eosinophilic, granular cytoplasm proliferating in tubular and papillary patterns, characteristic of pyloric gland adenoma. Scale bar: 100 μm. (**D**) High magnification view of Lesion III: tall columnar epithelium resembling gastric foveolar epithelium with inconspicuous nuclear atypia. Scale bar: 100 μm. (**E-a**–**E-c**) MUC5AC immunostaining of Lesions I, II, and III, respectively. All lesions are diffusely positive. Scale bars: 100 μm. (**F-a**–**F-c**) MUC6 immunostaining of Lesions I, II, and III, respectively. Lesion II shows positivity in the deeper glands, whereas Lesions I and III are negative. Scale bars: 100 μm. (**G-a**–**G-c**) Ki-67 immunostaining of Lesions I, II, and III, respectively. Lesions I and III show regular basal distribution of positive cells, whereas Lesion II shows irregular, scattered distribution. Scale bars: 100 μm.

**Figure 3:**
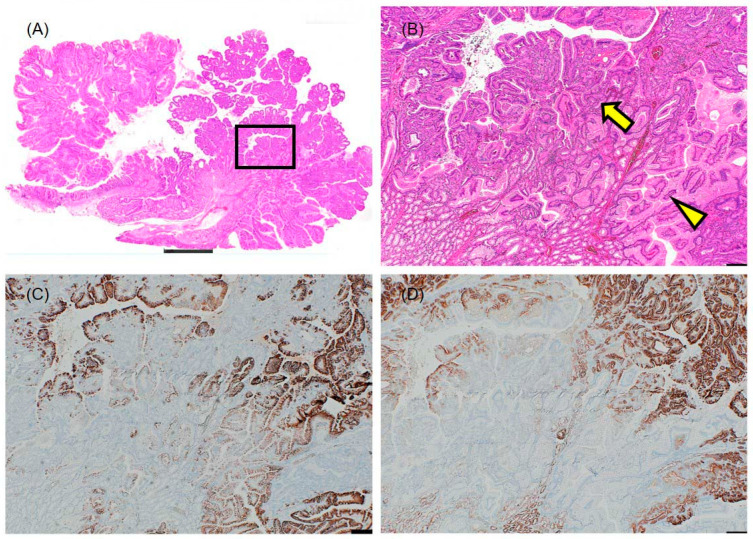
Transitional zone between Lesions I and II demonstrating histological continuity. (**A**) Loupe image indicating the location of the transitional zone (boxed area) between Lesion I (hyperplastic polyp) and Lesion II (pyloric gland adenoma). Scale bar: 2 mm. (**B**) High-magnification hematoxylin and eosin view of the transitional zone showing admixture of hyperplastic polyp components (pale columnar epithelium, arrowhead) and pyloric gland adenoma components (eosinophilic granular epithelium, arrow) within the same microscopic field. Scale bar: 200 μm. (**C**) MUC5AC immunostaining of the transitional zone. Both components are positive, confirming the shared gastric foveolar-type phenotype. Scale bar: 200 μm. (**D**) MUC6 immunostaining of the transitional zone. Positivity is observed in the adenomatous glands, while the hyperplastic component shows variable staining. Scale bar: 200 μm.

**Figure 4:**
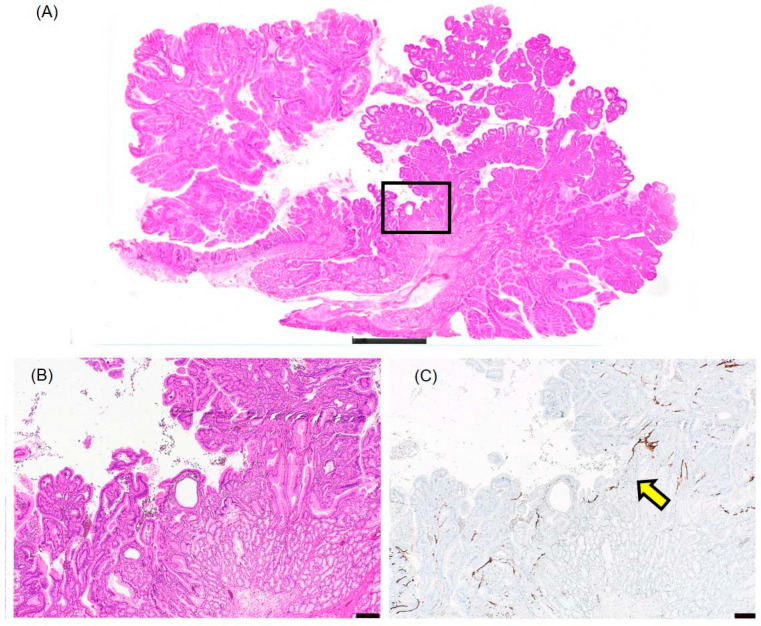
Intervening normal duodenal mucosa between Lesion III and Lesions I/II (slice “a”). (**A**) Loupe image showing the distribution of lesions in slice “a.” The boxed area indicates the intervening normal duodenal mucosa between the Lesion I/II complex and Lesion III. Scale bar: 2 mm. (**B**) Hematoxylin and eosin staining of the intervening normal duodenal mucosa showing preserved villous architecture. Scale bar: 400 μm. (**C**) CD10 immunostaining of the same field as (**B**). The normal mucosa shows positive brush border staining (arrow), confirming the intestinal phenotype. This finding establishes that Lesion III is a separate lesion from the Lesion I/II complex. Scale bar: 400 μm.

**Figure 5:**
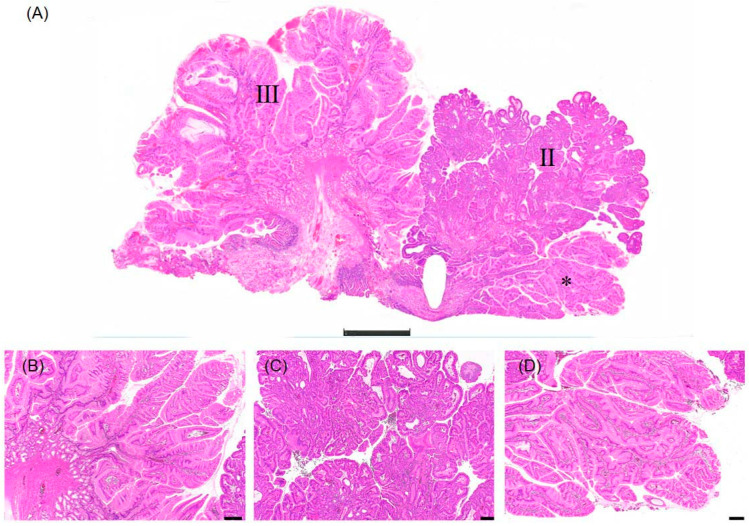
Histological findings of slice “b” containing Lesions II and III with extension of Lesion I. (**A**) Loupe image showing the distribution of Lesions II and III in slice “b.” The asterisk indicates an extension of Lesion I into this section. Scale bar: 2 mm. (**B**) High magnification view of Lesion III: tall columnar epithelium with inconspicuous nuclear atypia, consistent with hyperplastic polyp with gastric foveolar-type phenotype. Scale bar: 200 μm. (**C**) High magnification view of Lesion II: atypical cells with eosinophilic, granular cytoplasm, consistent with pyloric gland adenoma. Scale bar: 200 μm. (**D**) High magnification view of the Lesion I component (asterisk in (**A**)): columnar epithelial cells with pale cytoplasm, consistent with hyperplastic polyp with gastric foveolar-type phenotype. This finding confirms the presence of Lesion I extending into slice “b”. Scale bar: 200 μm.

An additional, smaller lesion measuring 10 × 5 mm was also resected from the adjacent mucosa. The entire specimen was serially sectioned and submitted for histological examination. No foci of adenocarcinoma or high-grade dysplasia were identified in any section. Histological examination revealed that the specimen contained a composite lesion with two contiguous components (Lesions I and II) and a separate lesion (Lesion III) (Figure 2, Figure 3, Figure 4 and Figure 5).

### 2.1. Lesions I and II: Contiguous Components of the Main Lesion

Lesion I (Figure 2A,B and Figure 5A,D) showed a dense proliferation of columnar epithelial cells with pale to slightly eosinophilic cytoplasm and mildly enlarged nuclei. Moderate nuclear atypia was observed without apparent structural atypia. Immunohistochemically, the proliferating glands were MUC5AC-positive and MUC6-negative (Figure 2E-a,F-a). Ki-67-positive cells were predominantly clustered in the proliferative zone, showing a regular basal distribution (Figure 2G-a). These findings were consistent with a hyperplastic polyp with a gastric foveolar-type phenotype.

Lesion II (Figure 2A,C and Figure 5A,C) showed atypical cells with eosinophilic, granular cytoplasm proliferating in tubular and papillary patterns, characteristic of PGA. Immunohistochemically, the superficial mucous glands were positive for MUC5AC, while the deeper glands were positive for MUC6 (Figure 2E-b,F-b). Small foci of pepsinogen-positive and proton pump-positive cells were observed, suggesting focal fundic gland differentiation, which was considered most likely reactive in nature. The brush border was not evident on CD10 staining. Ki-67-positive cells were few in number but showed a scattered, irregular distribution, suggesting loss of proliferative polarity (Figure 2G-b). Adjacent to the adenomatous component, hyperplastic changes with a gastric foveolar-type phenotype were also present, representing a transitional zone.

### 2.2. Transitional Zone Between Lesions I and II

Examination of the boundary between Lesions I and II revealed a transitional zone where hyperplastic polyp components and PGA components were admixed within the same microscopic field (Figure 3A–D). In this zone, the characteristic pale columnar epithelium of the hyperplastic component gradually transitioned into the eosinophilic, granular epithelium of the adenomatous component (Figure 3B). Immunohistochemical staining demonstrated that both components were positive for MUC5AC (Figure 3C), while MUC6 positivity was limited to the adenomatous glands (Figure 3D). This histological continuity strongly suggests that the hyperplastic and adenomatous components share a common origin and supports the concept of neoplastic transformation arising within a background of gastric-type hyperplasia.

### 2.3. Lesion III: Separate Hyperplastic Polyp

Lesion III (Figure 2A,D, Figure 4A and Figure 5A,B) was identified as a separate lesion, distinct from Lesions I and II. Normal duodenal mucosa with preserved villous architecture was present between Lesion III and the Lesion I/II complex. The presence of intervening normal mucosa was confirmed in slice “a” (Figure 4A–C). The normal duodenal mucosa showed intact brush border on CD10 staining (Figure 4C), confirming the intestinal phenotype of the intervening mucosa and establishing that Lesion III was indeed a separate lesion.

Histologically, Lesion III showed a moderately dense arrangement of tall columnar epithelium resembling gastric foveolar epithelium. The cells were aligned along the basement membrane with inconspicuous nuclear atypia (Figure 2D and Figure 5B). Immunohistochemically, the mucous glands were MUC5AC-positive and MUC6-negative (Figure 2E-c,F-c). Ki-67-positive cells were predominantly clustered in the proliferative zone, showing a regular basal distribution (Figure 2G-c). These findings were consistent with a hyperplastic polyp with a gastric foveolar-type phenotype, representing a separate lesion from the Lesion I/II complex.

### 2.4. Confirmation of Findings in Slice “b”

The histological findings from slice “a” were confirmed in slice “b” (Figure 5). Loupe imaging demonstrated Lesions II and III, along with an extension of Lesion I (Figure 5A, asterisk). High magnification views showed Lesion III with tall columnar epithelium (Figure 5B), Lesion II with characteristic PGA features (Figure 5C), and the Lesion I component with pale columnar epithelium consistent with a hyperplastic polyp with a gastric foveolar-type phenotype (Figure 5D).

### 2.5. Brunner’s Gland Hyperplasia and Background Findings

Brunner’s gland hyperplasia was observed extensively throughout the lesions, particularly in the basal portions of the specimen. The duodenal mucosa at the resection margin showed no evidence of GFM. No *H. pylori* organisms were detected in hematoxylin and eosin-stained or Giemsa-stained sections.

The immunohistochemical findings are summarized in Table 1.

All three lesions shared a gastric-type mucin phenotype (MUC5AC-positive), supporting a common histogenetic background. The irregular Ki-67 distribution in Lesion II reflects its neoplastic nature, contrasting the regular proliferative pattern observed in the non-neoplastic Lesions I and III.

Based on the histological and immunohistochemical findings, the final diagnoses were as follows:

Lesion I: Hyperplastic polyp with gastric foveolar-type phenotype (contiguous with Lesion II);
Lesion II: PGA mixed with gastric foveolar-type hyperplastic polyp;
Lesion III: Hyperplastic polyp with gastric foveolar-type phenotype (separate lesion).

Lesions I and II were interpreted as contiguous components of a single composite lesion, with histological continuity at the transitional zone. Lesion III was a separate lesion with intervening normal duodenal mucosa. All lesions shared a gastric-type mucin phenotype (MUC5AC-positive, CD10-negative).

### 2.6. Postoperative Course

The patient had an uneventful postoperative course. Follow-up endoscopy at 2 months post-ESD showed no evidence of residual or recurrent disease. Serological testing for *H. pylori* performed after ESD revealed a positive result (latex agglutination method, >16 U/mL), consistent with the endoscopic finding of atrophic gastritis.

## 3. Discussion

We report a rare case in which PGA and hyperplastic polyp components with gastric foveolar-type phenotype coexisted as contiguous lesions within a single ESD specimen from the duodenal bulb. The most significant finding was the histological continuity between the neoplastic (PGA) and non-neoplastic (hyperplastic polyp with gastric foveolar-type phenotype) components within the same lesion. To the best of our knowledge, this is the first report documenting the close coexistence and histological transition between PGA and hyperplastic polyp in the duodenum [6].

The key observation in this case was the transitional zone between Lesions I and II, where hyperplastic and adenomatous components were admixed within the same microscopic field. This finding provides direct morphological evidence that PGA can arise within a background of gastric-type hyperplasia, supporting the concept of a metaplasia–neoplasia sequence in the development of gastric-type duodenal neoplasms. Previous studies have reported that PGA arises in association with gastric heterotopia in approximately 23% of cases [6]; however, the histological transition from hyperplastic polyp to adenoma within a single lesion has not been previously documented. This transitional zone, where hyperplastic and adenomatous components coexist within a single microscopic field, represents a central teaching point for pathologists: it demonstrates that gastric-type hyperplastic and neoplastic lesions in the duodenum are not merely coincidental but may be part of a continuous histogenetic spectrum.

PGA is a rare neoplastic polyp that most commonly arises in the gastric corpus, often in association with autoimmune gastritis [2,3]. Extragastric sites, including the duodenum, have also been reported, with the duodenal bulb being the most frequent extragastric location [2,3]. PGA is characterized by tightly packed glands lined by cuboidal to low columnar epithelium with pale, ground-glass cytoplasm and is immunohistochemically positive for MUC5AC and MUC6 [2]. Pyloric gland metaplasia and gastric heterotopia have been proposed as precursor lesions of PGA [2,6]. Although PGA may appear histologically bland, it is a fully neoplastic lesion with documented malignant potential [6].

In contrast, GFM and hyperplastic polyps with gastric-type phenotype in the duodenum have traditionally been considered reactive changes, often associated with mucosal injury or *H. pylori* infection [1]. The histogenesis of gastric-type lesions in the duodenum has been linked to the “neo-G zone” hypothesis proposed by Kushima et al. [4]. According to this hypothesis, mucosal injury in the duodenum triggers a regenerative response in Brunner’s glands, leading to the formation of new generative cell zones that differentiate toward gastric foveolar-type epithelium. This metaplastic epithelium is thought to be more resistant to acid-peptic injury but may also be prone to neoplastic transformation.

Recent molecular studies have demonstrated that GFM and heterotopic gastric mucosa in the duodenum frequently harbor *GNAS* and *KRAS* mutations, which are also characteristic of PGA and gastric-type duodenal adenocarcinoma [5]. These findings suggest that gastric-type metaplasia may serve as a precursor lesion for gastric-type neoplasms in the duodenum. Importantly, even histologically benign gastric foveolar-type lesions may harbor these oncogenic mutations [7], supporting the concept of a metaplasia–neoplasia sequence in the development of gastric-type duodenal neoplasms. The present case provides morphological evidence that complements these molecular findings. While molecular analysis for GNAS and KRAS mutations was not performed in the present case, the morphological and immunohistochemical evidence presented here offers an independent line of support for the metaplasia–neoplasia sequence. Our findings demonstrate that the transition from gastric-type hyperplasia to neoplasia can be directly visualized at the tissue level, providing a structural framework that future molecular studies can build upon to elucidate the genetic mechanisms driving this progression.

A notable feature of this case is the development of large hyperplastic changes with a gastric foveolar-type phenotype. The mechanism underlying the development of such large lesions remains incompletely understood. Gastric foveolar-type hyperplasia arises as a hyperproliferative response to mucosal injury, characterized by excessive proliferation of foveolar cells accompanied by increased exfoliation [8]. Furthermore, Brunner’s gland proliferation has been shown to be enhanced beneath epithelium showing GFM, with higher MIB-1 labeling indices observed at sites of surface erosion and beneath metaplastic epithelium [9]. In the present case, extensive Brunner’s gland hyperplasia was observed throughout the lesions, supporting the hypothesis that repeated or chronic mucosal injury may have contributed to the development of these large gastric-type lesions through a coordinated proliferative response involving both surface epithelium and Brunner’s glands. Furthermore, Brunner’s glands normally open into the crypts, but during the tissue repair process following erosion or ulceration, they may open directly into the lumen, accompanied by transformation of the overlying surface epithelium into gastric foveolar-type epithelium. Brunner’s gland hyperplasia, by forming elevated lesions, is inherently susceptible to surface erosion; larger lesions are more likely to undergo repeated cycles of mucosal damage and repair. This process would promote progressive gastric foveolar-type metaplasia of the overlying epithelium, ultimately leading to the formation of hyperplastic polyps with a gastric foveolar-type phenotype. Thus, a direct relationship between Brunner’s gland hyperplasia and the development of hyperplastic polyps in the present case cannot be excluded.

In Lesion II (PGA), small foci of pepsinogen-positive and proton pump-positive cells were observed, suggesting focal fundic gland differentiation. This finding is most likely reactive in nature, possibly reflecting the multipotent differentiation capacity of the neoplastic cells or the influence of the surrounding microenvironment.

Another notable finding in this case is the presence of Lesion III, a separate hyperplastic polyp with a gastric foveolar-type phenotype, which was clearly separated from the Lesion I/II complex by intervening normal duodenal mucosa. The coexistence of neoplastic (PGA in Lesion II) and non-neoplastic (hyperplastic polyps in Lesions I and III) lesions with a shared gastric-type phenotype within a confined area suggests the possibility of “field carcinogenesis” or “field change” in the duodenal bulb. However, given that Lesion III was a non-neoplastic hyperplastic polyp without evidence of dysplasia, this finding may more accurately reflect a “field of metaplastic susceptibility” rather than true field cancerization. The affected duodenal mucosa may be broadly predisposed to gastric-type metaplasia as a result of chronic mucosal injury, with neoplastic transformation occurring only in a subset of metaplastic foci. This distinction has important clinical implications: patients with multifocal gastric-type lesions in the duodenum may be at risk of multicentric lesion development and should be considered for long-term endoscopic surveillance. Further molecular studies would be needed to clarify whether these separate lesions share common genetic alterations or arise independently within a predisposed mucosal field.

From a clinical and diagnostic perspective, this case highlights the limitations of preoperative biopsy in characterizing heterogeneous gastric-type duodenal lesions. The preoperative biopsy in this case was diagnosed as PGA, which was indeed an accurate diagnosis for the sampled portion of the lesion. However, it captured only one aspect of a far more complex lesion. The coexistence of a contiguous hyperplastic polyp component with histological continuity to the adenoma, as well as a separate hyperplastic polyp, was entirely unrecognized on biopsy and only became apparent after complete resection and thorough histopathological examination. This discrepancy illustrates that even when a biopsy diagnosis is correct, it may not reflect the full histological diversity of heterogeneous duodenal lesions [10]. The composite nature of the lesion—with its admixture of neoplastic and non-neoplastic components—could only be appreciated in the resected specimen. Therefore, this case reinforces the importance of complete resection for large or endoscopically heterogeneous gastric-type lesions in the duodenum, not only for therapeutic purposes but also for accurate pathological characterization. Pathologists should be aware that gastric-type duodenal lesions may harbor a broader spectrum of histological changes than what is captured by biopsy alone.

This study has some limitations. First, although *H. pylori* serology was positive after ESD, the endoscopic finding of atrophic gastritis suggests chronic infection, which may have contributed to the development of gastric-type lesions in the duodenum. Second, molecular analysis for *GNAS* and *KRAS* mutations was not performed. Genetic profiling could provide additional insights into the clonal relationship between the neoplastic and non-neoplastic components in this case. Third, although no *H. pylori* organisms were detected on hematoxylin and eosin-stained or Giemsa-stained sections, this does not exclude prior or current infection, particularly in the setting of atrophic gastritis, where organism density may be very low. Although Giemsa staining has limited sensitivity in the setting of atrophic gastritis with low organism density, endoscopic evaluation of *H. pylori* infection status was clinically prioritized over additional immunohistochemistry. Future studies utilizing laser capture microdissection of the transitional zone, followed by mutational analysis, would be valuable to confirm the clonal relationship between the neoplastic and non-neoplastic components. Nevertheless, our morphological and immunohistochemical findings provide compelling evidence for a histogenetic link between these lesions.

## 4. Conclusions

We report the first case demonstrating histological continuity between PGA and a hyperplastic polyp with a gastric foveolar-type phenotype within a single duodenal lesion. The transitional zone, where adenomatous and hyperplastic components were admixed within the same microscopic field, provides direct morphological evidence supporting the concept of a metaplasia–neoplasia sequence in the development of gastric-type duodenal neoplasms. Additionally, the presence of a separate hyperplastic polyp with the same phenotype, separated by normal duodenal mucosa, suggests a field change in the duodenal bulb. Notably, although the preoperative biopsy correctly diagnosed PGA, the composite nature of the lesion—including a contiguous hyperplastic polyp component and histological transition between the two—was only revealed by complete resection. This finding underscores the limitations of biopsy sampling in capturing the full histological spectrum of heterogeneous gastric-type duodenal lesions. Complete endoscopic resection should be considered for large or endoscopically heterogeneous gastric-type lesions in the duodenum, both for therapeutic and diagnostic purposes. Indeed, the composite nature of the present lesion—comprising neoplastic and non-neoplastic components with histological continuity—could only be recognized through complete resection and thorough histopathological examination. This case reinforces the principle that for heterogeneous duodenal lesions, complete resection is essential for accurate diagnosis, as biopsy alone may fail to represent the full histological diversity of such complex lesions.

## Figures and Tables

**Table 1:** Immunohistochemical Profile of the Lesions in the Present Case.

Marker	Lesion I	Lesion II	Lesion III	Interpretation
MUC5AC	+	+	+	Gastric foveolar type
MUC6	−	+	−	Pyloric gland type (Lesion II)
CD10	−	−	−	Non-intestinal type
Pepsinogen	−	±	−	Focal fundic diff. (Lesion II)
Proton pump	−	±	−	Focal fundic diff. (Lesion II)
Ki-67 distribution	Regular, basal	Irregular, scattered	Regular, basal	Loss of polarity (Lesion II)

Note: Immunohistochemical results: + positive, − negative, ± rare positive cells. Primary antibodies used: MUC5AC (clone MRQ-19; Roche), MUC6 (clone MRQ-20; Roche), CD10 (clone SP67; Roche), pepsinogen I (clone 8003; BIO-RAD), proton pump H,K-ATPase (MBL), Ki-67 (clone 30-9; Roche), and p53 (data not shown; negative in all lesions).

## Data Availability

The original contributions presented in this study are included in the article. Further inquiries can be directed to the corresponding author.

## Abbreviations

The following abbreviations are used in this manuscript:

PGA	Pyloric gland adenoma
GFM	Gastric foveolar metaplasia
NBI	Narrow-band imaging
ESD	Endoscopic submucosal dissection
